# Decreased miR-106a inhibits glioma cell glucose uptake and proliferation by targeting SLC2A3 in GBM

**DOI:** 10.1186/1471-2407-13-478

**Published:** 2013-10-14

**Authors:** Dong-Wei Dai, Qiong Lu, Lai-Xing Wang, Wen-Yuan Zhao, Yi-Qun Cao, Ya-Nan Li, Guo-Sheng Han, Jian-Min Liu, Zhi-Jian Yue

**Affiliations:** 1 Department of Neurosurgery, Changhai Hospital, Second Military Medical University, 168 Changhai Road, Shanghai 200433, China; 2 Department of Laboratory Medicine, Changhai Hospital, Second Military Medical University, Shanghai China

**Keywords:** miR-106a, SLC2A3, Cell proliferation, Glucose uptake, GBM

## Abstract

**Background:**

MiR-106a is frequently down-regulated in various types of human cancer. However the underlying mechanism of miR-106a involved in glioma remains elusive.

**Methods:**

The association of miR-106a with glioma grade and patient survival was analyzed. The biological function and target of miR-106a were determined by bioinformatic analysis and cell experiments (Western blot, luciferase reporter, cell cycle, ntracellular ATP production and glucose uptake assay). Finally, rescue expression of its target SLC2A3 was used to test the role of SLC2A3 in miR-106a-mediated cell glycolysis and proliferation.

**Results:**

Here we showed that miR-106a was a tumor suppressor miRNA was involved in GBM cell glucose uptake and proliferation. Decreased miR-106a in GBM tissues and conferred a poor survival of GBM patients. SLC2A3 was identified as a core target of miR-106a in GBM cells. Inhibition of SLC2A3 by miR-106a attenuated cell proliferation and inhibited glucose uptake. In addition, for each biological process we identified ontology-associated transcripts that significantly correlated with SLC2A3 expression. Finally, the expression of SLC2A3 largely abrogated miR-106a-mediated cell proliferation and glucose uptake in GBM cells.

**Conclusions:**

Taken together, miR-106a and SLC2A3 could be potential therapeutic approaches for GBM.

## Background

MicroRNAs (miRNAs) have been demonstrated to play critical roles in the development and progression of cancer by blocking target mRNA translation [1,2]. Deregulated miRNAs was identified as oncogenic miRNAs or tumor suppressor miRNAs in glioma [3-5]. MiR-106a was significantly down-regulated in human high grade glioma tissues [6]. MiR-106a suppressed cell proliferation and induced cell apoptosis in glioma cells by targeting E2F1 [7]. It was also revealed that miR-106a increased p53 expression via E2F1 inhibition, whereas the effect of miR-106a on the proliferation of glioma cells was independent of p53 status. However, the functional role and underlying mechanism of miR-106a involved in glioma still remain unknown and demand further investigations.

In our present work, we showed the function and mechanism of miR-106a involved in glioblastoma (GBM). MiR-106a inhibited GBM cell proliferation and glucose uptake by repressing SLC2A3. Decreased miR-106a and increased SLC2A3 indicated a poor survival of GBM patients. Thus, miR-106a and SLC2A3 could be potential therapeutic approaches for GBM treatment.

## Methods

### Clinical sample collection

Human tissues used in this study were obtained in 2012 from Changhai Hospital, Second Military Medical University in China, including 3 normal brain tissues, 6 grade II glioma tissues, 6 grade III glioma tissues and 7 grade IV glioma (GBM) tissues in the supplementary material (Additional file 1: Table S1). Tumor tissue samples were obtained by surgical resection. Normal brain tissues were obtained during surgery for severe traumatic brain injury. Specimens were snap-frozen in liquid nitrogen. Patients were selected for the study if their diagnoses were established histologically according to the World Health Organization classification guidelines by two neuropathologists. The collection and use of the patient samples were reviewed and approved by the Ethics Committee Review Board of Changhai Hospital, and written informed consent from all patients were appropriately obtained.

### In silico analysis

The miRNA and mRNA expression profiles containing 465 GBMs with complete survival information were obtained from TCGA database. Also the mRNA expression profilings containing 67 GBMs with complete survival information were obtained from GEO database (http://www.ncbi.nlm.nih.gov/geo/query/acc.cgi?acc=GSE4290). Using the AmiGO tool [8] of the Gene Ontology, project lists of transcripts associated with the biological processes proliferation (GO:0008283) and glycolysis (GO:0006096) were obtained. Subsequently the microarray dataset was queried for the genes in each of these ontologies. Samples were sorted on SLC2A3 expression level and the expression levels scaled on a gene by gene basis for genes significantly correlating with SLC2A3 expression (P < 0.001) were plotted as a heatmap.

### Cell culture and transfection

U251 and LN229 GBM cells were maintained in DMEM medium supplemented with 10% fetal bovine serum. MiR-106a, and negative control oligonucleotides were purchased from GenePharma (Shanghai, China). For expression plasmid construct, wild-type SLC2A3 cDNA sequence without 3′UTR was selected and cloned into Pgenesil-1 vector. Cells were transfected using Lipofectamine 2000 (Invitrogen) at the time of 50-60% confluence. 48 h after transfection, cells were harvested for further studies.

### Quantitative RT -PCR

Real-time quantification of hsa-miR-106a was performed by stem-loop RT-PCR. All the primers of miRNAs for TaqMan miRNA assays were purchased from GenePharma Co., Ltd. (Shanghai, China). Human SLC2A3 (forward)/ (reverse): 5′TCCCCTCCGCTGCTCACTATTT3′ and 5′ATCTCCATGA CGCCGTCCTTTC3′. GAPDH (forward)/ (reverse): 5′-GTCGGAGTCAACGGATT-3′; 5′-AAGCTTCCCGTTCTCAG-3′. Real-time PCR was performed according to the manufacturer’s instructions. All experiments were performed using biological triplicates and experimental duplicates. The relative expression was calculated via the 2-ΔΔCt method.

### MTT assay

Cells were plated at 10^4^ cells per well in 96-well plates with six replicate wells. After transfection as described previously, 20 μl of MTT (5 g/L, Sigma) was added into each well at each day of 4 consecutive days after treatment and the cells were incubated for additional 4 h. The supernatant was then discarded. 200 μl of DMSO was added to each well to dissolve the precipitate. Optical density (OD) was measured at the wave length of 550 nm. The data were presented as mean ± SD, which were derived from triplicate samples of at least three independent experiments.

### Cell cycle analysis

Cells were washed with PBS, fixed with 70% ethanol for at least 1 h. After extensive washing, the cells were suspended in PBS containing 50 μg/mL PI and 50 μg/ml RNase A and incubated for 1 h at room temperature, and analyzed by FACScan (Becton Dickinson). Cell cycle analysis was performed by ModFit software. Experiments were performed in triplicate. Results were presented as% of cell in a particular phase.

### Intracellular ATP production and glucose uptake assay

Intracellular ATP levels were measured using ATP Bioluminescence Assay Kit (Roche Applied Science). Briefly, 5 × 105 cells were lysed with boiling lysis reagent and supernatant was collected. Fifty microliters of diluted sample were mixed with 50 μL of luciferin/luciferase reagents. Luminescence was measured using Luminoskan Ascent (Thermo Scientific). For glucose uptake assay, 3 × 105 cells were incubated in the presence of 20 μmol/L of 2-NBDG (Invitrogen) for 2 hours. The cells were resuspended in a cold growth medium and stained with propidium iodide. Samples were maintained on the ice and analyzed by flow cytometry (Becton Dickinson).

### Western blot analysis

Equal amounts of protein per lane were separated by 8% SDS-polyacrylamide gel and transferred to PVDF membrane. The membrane was blocked in 5% skim milk for 1 h and then incubated with a specific antibody for 2 h. The antibodies used in this study were: antibodies to SLC2A3 (Santa Cruz). The antibody against GAPDH (Santa Cruz) was used as control. The specific protein was detected by using a SuperSignal protein detection kit (Pierce). The band density of specific proteins was quantified after normalization with the density of GAPDH.

### Luciferase reporter assay

The human SLC2A3 3′UTR was amplified and cloned into the XbaI site of the pGL3-control vector (Promega), downstream of the luciferase gene, to generate the plasmids WT-SLC2A3-3′UTR in the supplementary material (Additional file 2: Figure S1). MUT- SLC2A3-3′UTR plasmids were generated from WT-SLC2A3-3′UTR by deleting the binding site for miR-106a “CACUUU”. For the luciferase reporter assay, cells were cultured in 96-well plates, transfected with the plasmids and miR-106a using Lipofectamine 2000. 48 h after transfection, luciferase activity was measured using the Luciferase Assay System (Promega).

### Statistical analysis

Statistics was determined by ANOVA, t test, Pearson correlation or Kaplan-Meier analysis. Statistical significance was determined as P < 0.05.

## Results

### Decreased miR-106a confers a poor prognosis in GBMs

**Figure 1:**
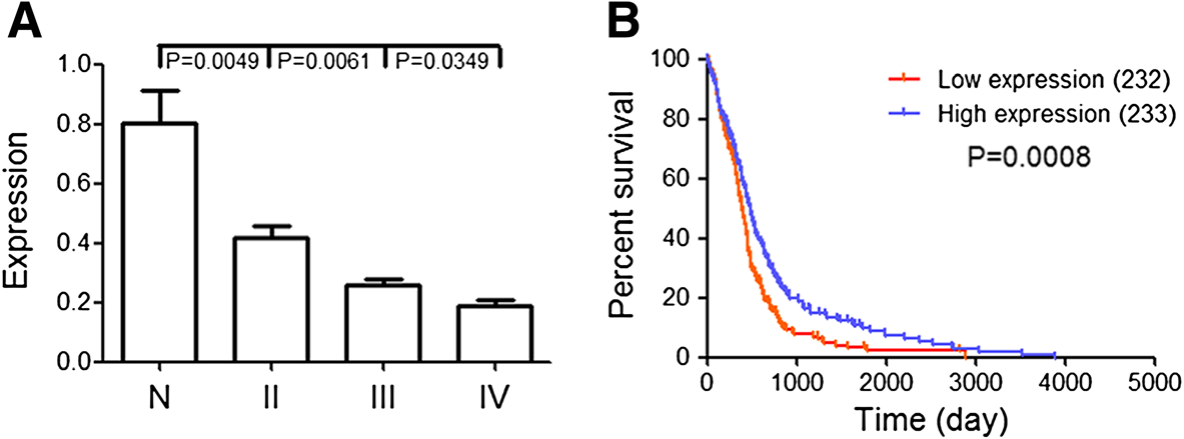
**Decreased miR-106a confers a poor prognosis in GBMs. (A)** Real time PCR was employed to measure the expression of miR-106a in glioma specimens and normal tissues. **(B)** Kaplan-Meier survival curves for miR-106a expression in GBM of TCGA data.

To explore miR-106a expression in gliomas, we examined 19 human glioma specimens and 3 normal brain tissues using Real time PCR. As shown in Figure 1A, the levels of miR-106a decreased markedly in glioma in comparison to normal tissues (P < 0.01). Further, miR-106a was significantly down-regulated in high grade gliomas compared to that of low grade gliomas (P < 0.05). We also found miR-106a expression was significantly lower in U87 and LN229 cells (Additional file 3: Figure S2).

Next we investigated the correlation between miR-106a expression and overall survival through Kaplan-Meier survival curve analysis with a log-rank comparison. In TCGA data, we chose 465 GBMs with complete survival data for further analysis (Figure 1B). GBM samples expressing lower level of miR-106a were associated with decreased survival relative to those with higher level (P = 0.008). These data indicate that the cases with lower miR-106a expression have a markedly worse outcome.

### SLC2A3 is a direct target of miR-106a

**Figure 2:**
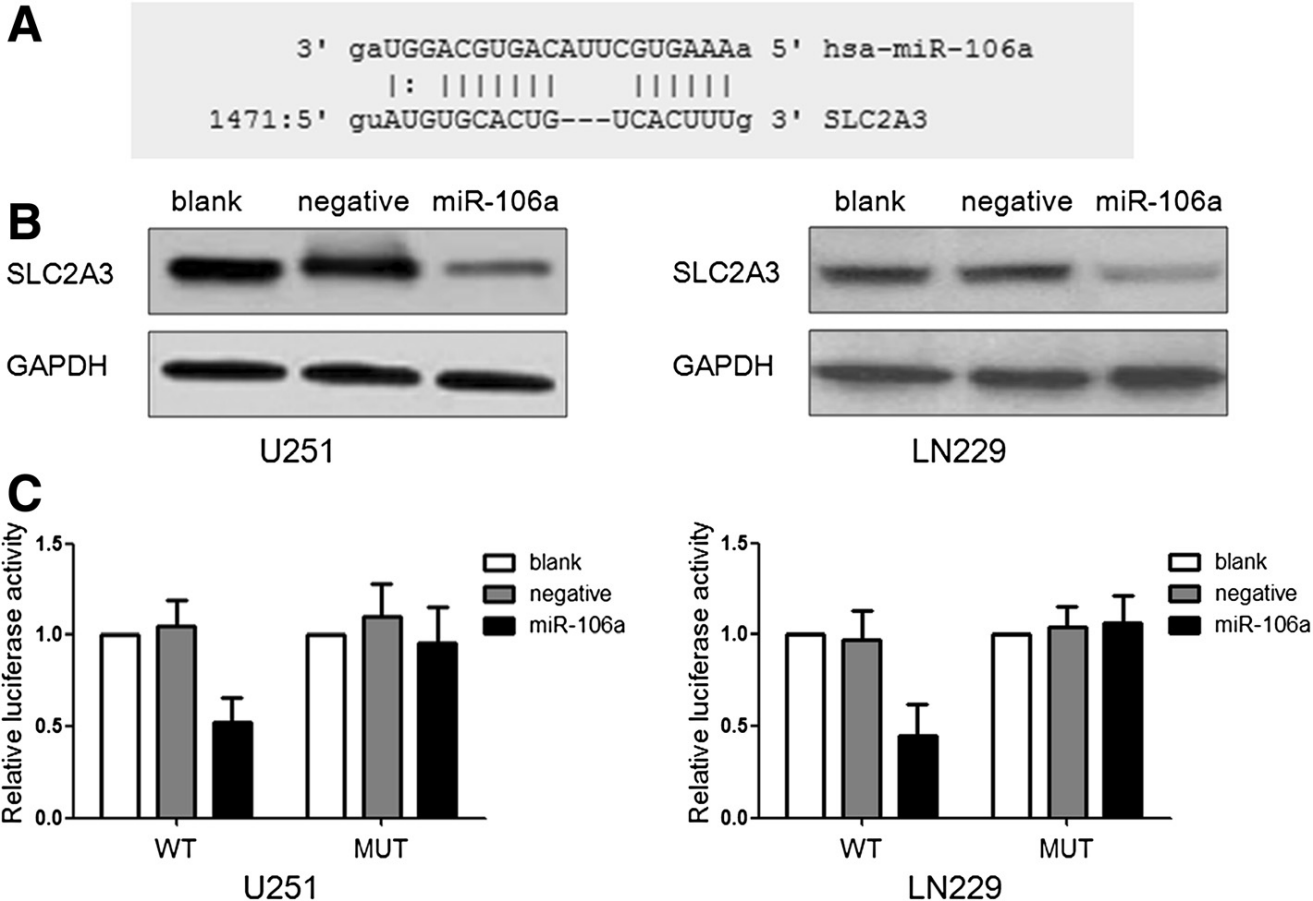
**SLC2A3 is a target gene of miR-106a. (A)** A schematic representation showing the putative target site for miR-106a in SLC2A3 mRNA 3′UTR. **(B)** Cells were transfected with miR-106a, and the expression of SLC2A3 was analyzed by Western blot. The expression of GAPDH was used as a loading control. **(C)** Luciferase constructs were transfected into cells transduced with miR-106a. Luciferase activity was determined 48 h after transfection. The ratio of normalized sensor to control luciferase activity is shown.

miRNA target bioinformatics analysis showed that SLC2A3 contained the highly conserved putative miR-106a binding sites (Figure 2A). To determine whether SLC2A3 was directly regulated by miR-106a, Western blot analysis and luciferase reporter assay were performed. Western blot analysis showed that a marked reduction of SLC2A3 expression was observed after over-expression of miR-106a both in U251 and LN229 cells (Figure 2B). Further, we created WT-SLC2A3-3′UTR, and MUT-SLC2A3-3′UTR plasmids. Reporter assay revealed that miR-106a induced a significant reduction of luciferase activity of WT-SLC2A3-3′UTR plasmid in U251 and LN229 cells, but without significant changes in luciferase activity of MUT-SLC2A3-3′UTR (Figure 2C).

### SLC2A3 is inversely correlated with miR-106a and GBM survival

**Figure 3:**
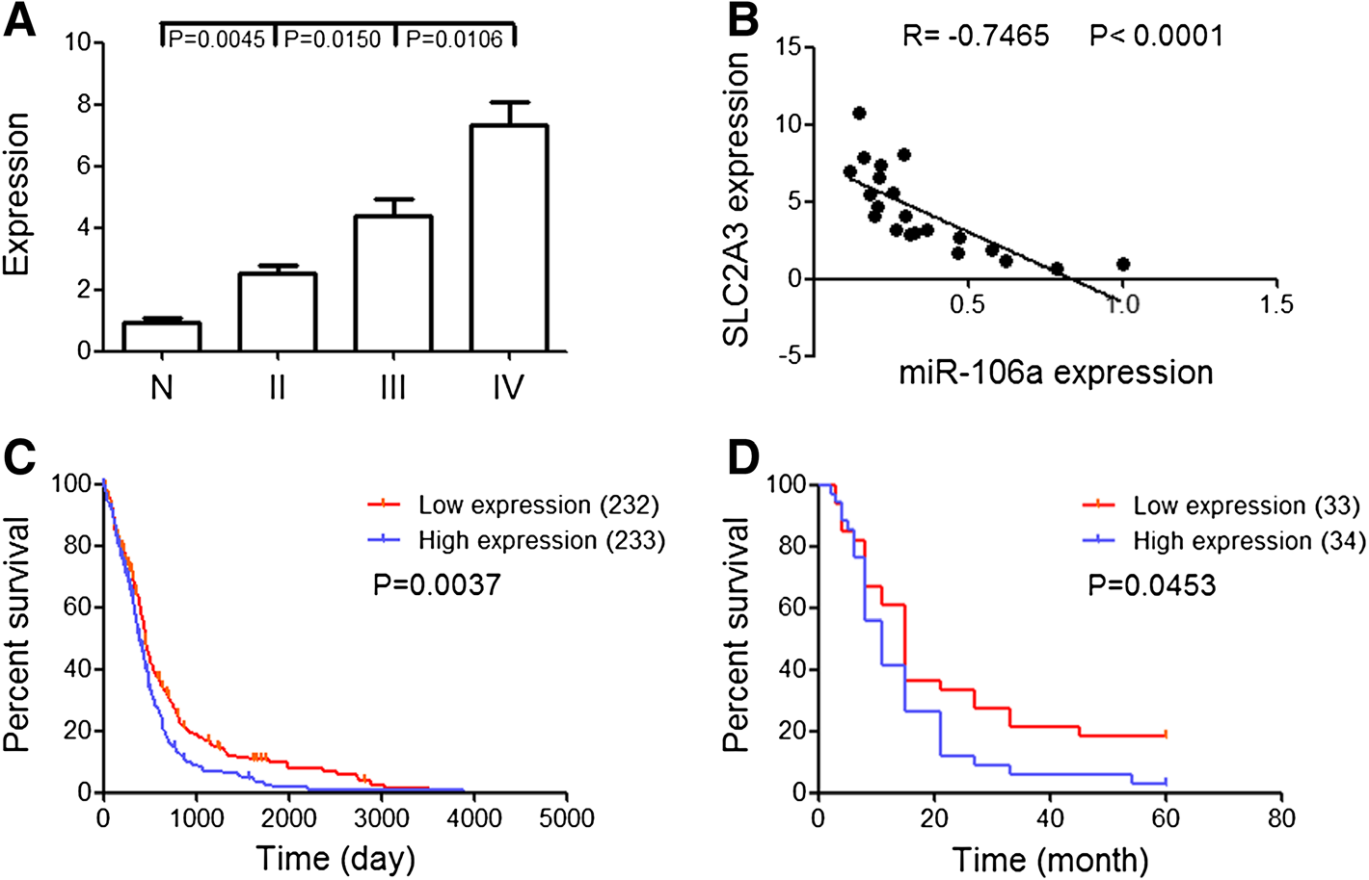
**SLC2A3 is inversely correlated with miR-106a and GBM survival. (A)** Expression levels of SLC2A3 in gliomas were measured by Real time PCR. **(B)** Inverse correlation of miR-106a expression with SLC2A3 expression in glioma tissues by Pearson correlation analysis. **(C, D)** Kaplan-Meier survival curves for SLC2A3 expression in GBM of TCGA data and GSE4290 data.

We further explored the correlation between miR-106a and SLC2A3 expression in gliomas. Real time PCR showed that the levels of SLC2A3 increased markedly in high grade gliomas in comparison to low grade gliomas and normal tissues (P < 0.05) (Figure 3A). Correlation analysis revealed that a significant negative correlation existed between miR-106a and SLC2A3 expression (R = −0.7465, P < 0.0001) (Figure 3B). Also we found that miR-106a expression was negatively associated with SLC2A3 expression in 465 GBMs of TCGA (R = −0.1392, P = 0.0026). These data suggest that SLC2A3 is a direct target of miR-106a in gliomas.

Also we explored the correlation between SLC2A3 expression and GBM survival. In 465 GBMs of TCGA, the samples with a higher SLC2A3 level had a poor prognosis (P = 0.0037) (Figure 3C). To further confirm this result, we examined it in another independent cohort (GSE4290). Kaplan-Meier survival curve analysis showed that a statistically significant correlation was observed between the survival and the expression levels of SLC2A3 (P = 0.0453) (Figure 3D). These data indicate that the SLC2A3 high positive cases have a worse outcome.

### MiR-106 inhibits glioma cell proliferation

**Figure 4:**
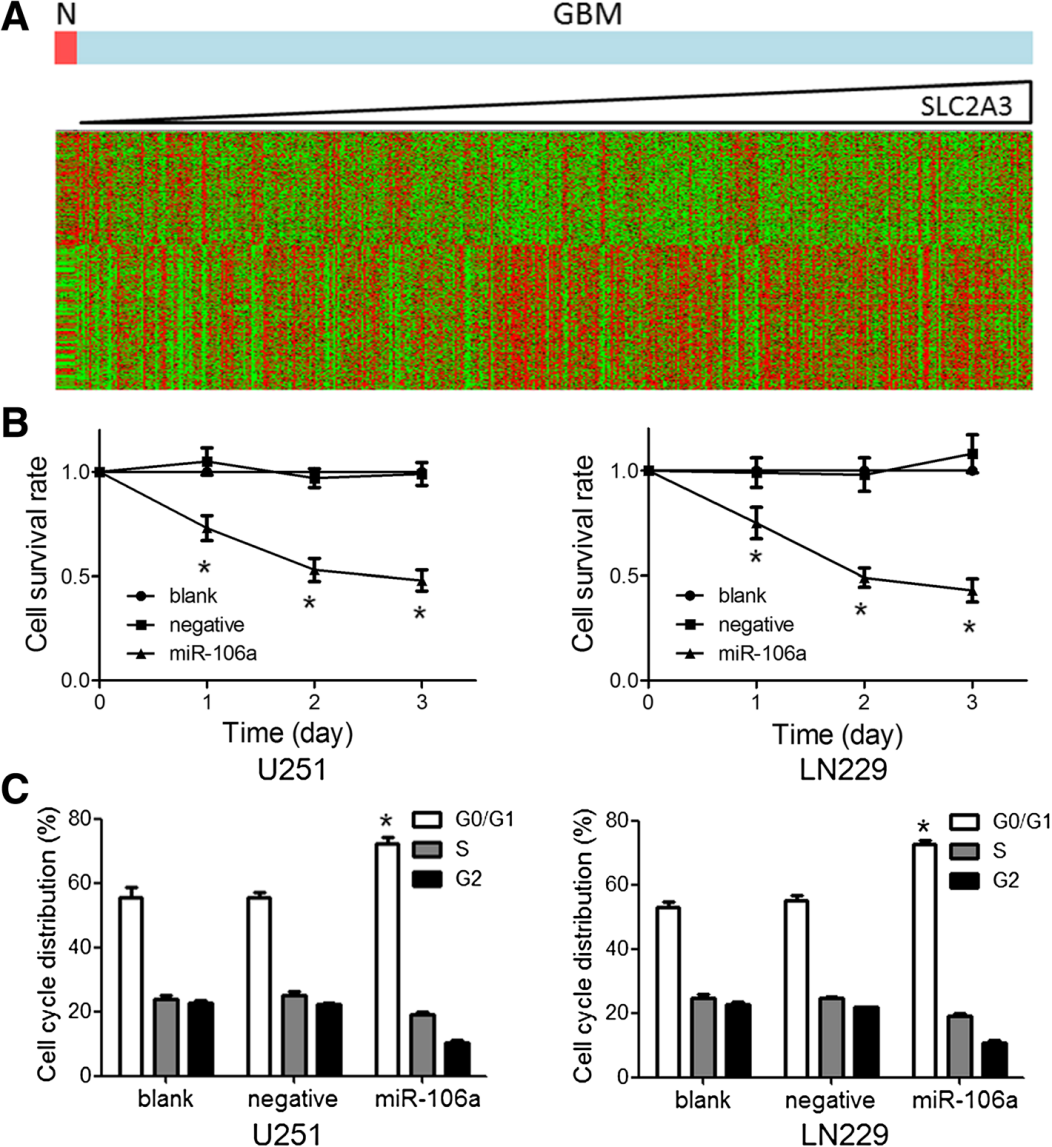
**MiR-106 inhibits glioma cell proliferation in vitro. (A)** In silico analysis of SLC2A3 mRNA expression and the correlation to proliferation-related mRNAs. Heatmap of gene expression of proliferation-related genes in patients was sorted by level of SLC2A3 expression (columns). **(B)** MTT assay displayed that cells treated with miR-106a proliferated at a significantly lower rate than control groups after transfection. **(C)** After 48 h treatment, cells were harvested and performed by cell cycle assay. Data were presented as the mean of triplicate experiments.

To determine SLC2A3-mediated effects of miR-106a on glioma cell proliferation, we first analyzed which genes were associated with cell proliferation and correlated with SLC2A3 expression in glioma. First, by analyzing the genes linked to the proliferation gene ontology as determined by AmiGO [8], 836 genes showed a clear correlation (P < 0.001) with SLC2A3 expression in GBM of TCGA (Figure 4A). Interestingly, GBM samples with normal SLC2A3 expression levels also showed a similar gene expression pattern with normal tissues. Next, miR-106a induction significantly reduced cellular proliferation in U251 and LN229 cells (Figure 4B). Furthermore, miR-106a treated cells represented significant ascends in G0/G1 phase in comparison to untreated cells (Figure 4C).

### MiR-106a inhibits glioma cell glucose uptake

**Figure 5:**
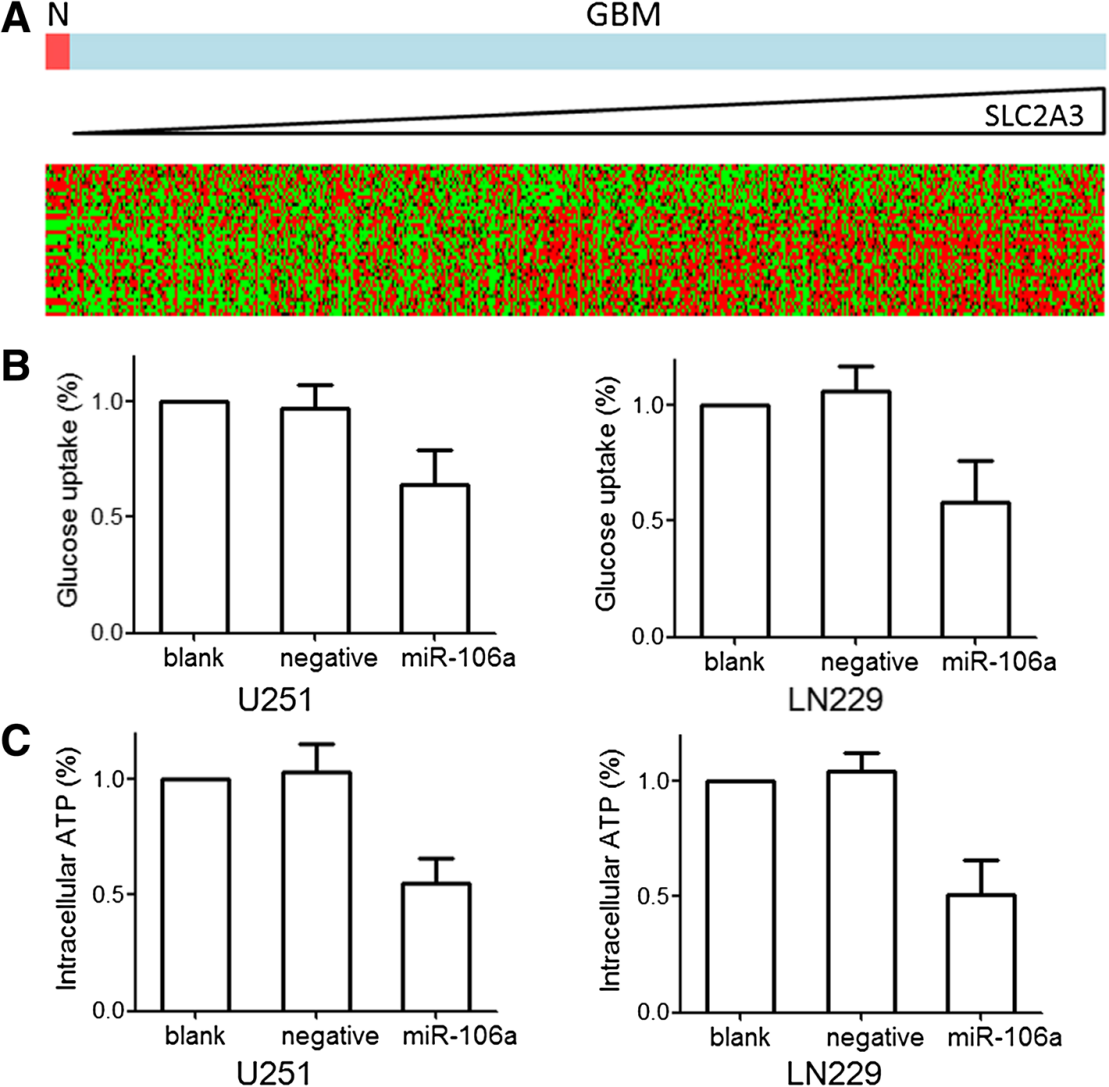
**MiR-106a inhibits glioma cell glucose uptake in vitro. (A)** In silico analysis of SLC2A3 mRNA expression and the correlation to apoptosis-related mRNAs. Heatmap of gene expression of glycolysis-related genes in patients was sorted by level of SLC2A3 expression (columns). **(B and C)** After 48 h treatment of miR-106a or SLC2A3 siRNA, cells were harvested and glucose uptake and intracellular ATP production assays were performed. Data were presented as the mean of triplicate experiments.

Since it had been reported that SLC2A3 could regulate cell glucose uptake [9]. We analyzed which genes belonged to the glycolysis gene ontology correlated with SLC2A3 expression. A significant correlation between the expression of 76 genes associated with cell glycolysis and SLC2A3 expression was observed (Figure 5A). To determine whether miR-106a up-regulation affected glioma cell glycolysis, glucose uptake and intracellular ATP production were evaluated. Over-expressed miR-106a resulted in a significant decrease of glucose uptake and ATP production in U251 and LN229 cells (Figure 5B and C).

### Functional role of SLC2A3 in miR-106a-mediated cell glycolysis and proliferation

**Figure 6:**
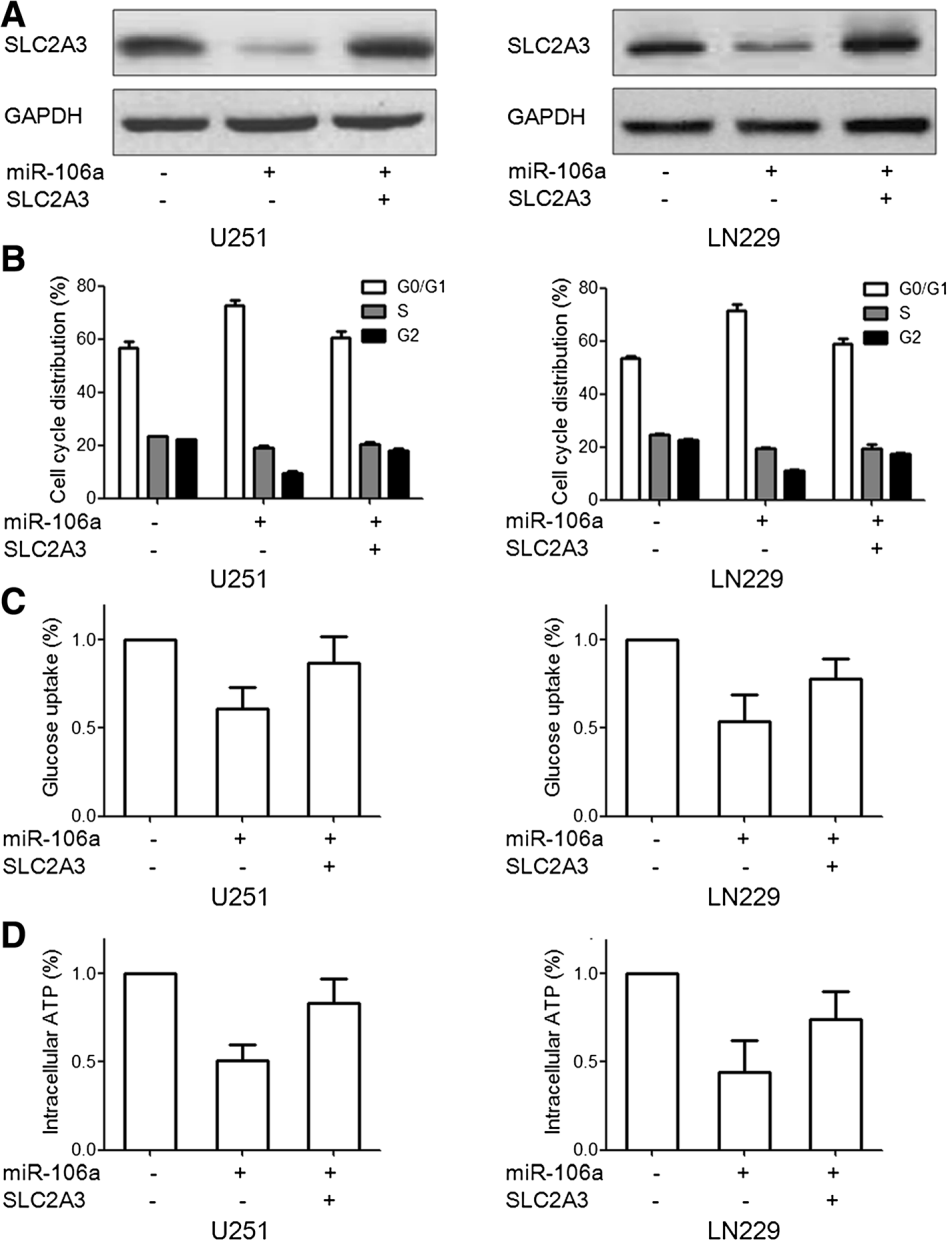
**Expression of SLC2A3 abrogates miR-106a-mediated glycolysis and proliferation. (A)** Cells were transfected with SLC2A3 plasmid (without the 3′UTR) and miR-106a. SLC2A3 protein level was detected by Western blot assay. GAPDH protein was regarded as endogenous normalizer. **(B-D)** Cells were transfected with SLC2A3 plasmid (without the 3′UTR) and miR-106a. Cell cycle, glucose uptake and intracellular ATP production assays were performed respectively. Data were expressed as mean ± SD of 3 independent experiments.

Having demonstrated SLC2A3 as a target of miR-106a, we next examined the importance of SLC2A3 in miR-106a -mediated cell glycolysis and proliferation. First we constructed SLC2A3 expression plasmid without 3′UTR. Western blot assay showed that transfection with SLC2A3 plasmid without 3′UTR abrogated SLC2A3 expression targeted by miR-106a (Figure 6A). By further transfecting with SLC2A3 without 3′UTR and miR-106a, expression of SLC2A3 largely overrode the effect of miR-106a on cell glycolysis and proliferation (Figure 6B-D). These findings suggest that SLC2A3 is a major functional target of miR-106a involved in glioma cell glycolysis and proliferation.

## Discussion

Recent studies have shown that miR-106a has played important roles in the development and progression of human tumors. MiR-106a was up-regulated in gastric carcinoma [10], colorectal cancer [11] and mantle cell lymphoma [12], whereas down-regulated in glioma. In this study, we found that miR-106a was a tumor suppressor miRNA associated with GBM outcome, consistent with previous data [7,13]. Over-expression of miR-106a, down-regulated SLC2A3 expression via targeting 3′UTR of SLC2A3, resulted in cell proliferation and cell glycolysis inhibition in GBM cells. To our knowledge, this is the first time to show the glycolysis function of miR-106a.

SLC2A3, also named as glucose transporter 3 (GLUT3), has a high affinity for glucose, and is recognized as an oncogene in several human cancers [14-17]. In oral squamous cell carcinoma, positive cell membrane SLC2A3 protein expression was associated with advanced clinic-staging of tumors and positive expression of SLC2A3 was also associated with unfavorable free-disease survival [18]. Other data showed that both endometrial and breast poorly differentiated tumors had significantly higher GLUT1 and GLUT3 expressions than well-differentiated tumors [19]. In our study, we also showed that SLC2A3 was over-expressed in high grade glioma tissues, and repression SLC2A3 abrogated miR-106a-mediated cell proliferation and glucose uptake in GBM cells. These data suggest that higher SLC2A3 expression in glioma is associated closely with an aggressive and poor prognostic phenotype.

## Conclusion

In conclusion, we have shown that miR-106a is one of the tumor suppressor miRNAs and SLC2A3 is a novel and critical target of miR-106a in GBM. These results suggest that miR-106a and SLC2A3 might be useful as a potential therapeutic target for GBM and more in-depth analyses are required in the future.

## Competing interests

The authors declare that they have no competing interests.

## Authors’ contributions

DD and QL carried out studies, data analysis and drafted the paper; LW and WZ participated in data analysis; YC, YL and GH participated in the design of the study and statistical analysis; JL and YZ participated in design and cooperated in the draft and final revision of the paper. All authors read and approved of the final manuscript.

## Pre-publication history

The pre-publication history for this paper can be accessed here:

http://www.biomedcentral.com/1471-2407/13/478/prepub

## Supplementary Material

Additional file 1: Table S1 WHO grade,Sex and Age of 19 glioma patients. Click here for file

Additional file 2: Figure S1 The XbaI site of the pGL3-control vector (Promega). Click here for file

Additional file 3: Figure S2 miR-106a expression was significantly lower in U87 and LN229 cells. Click here for file
