# Supplementary figures and images for: Decreased miR-106a inhibits glioma cell glucose uptake and proliferation by targeting SLC2A3 in GBM
Source: BMC Cancer. 2013 Oct 14;13:478. doi: 10.1186/1471-2407-13-478 (PMC3853007; doi:10.1186/1471-2407-13-478)

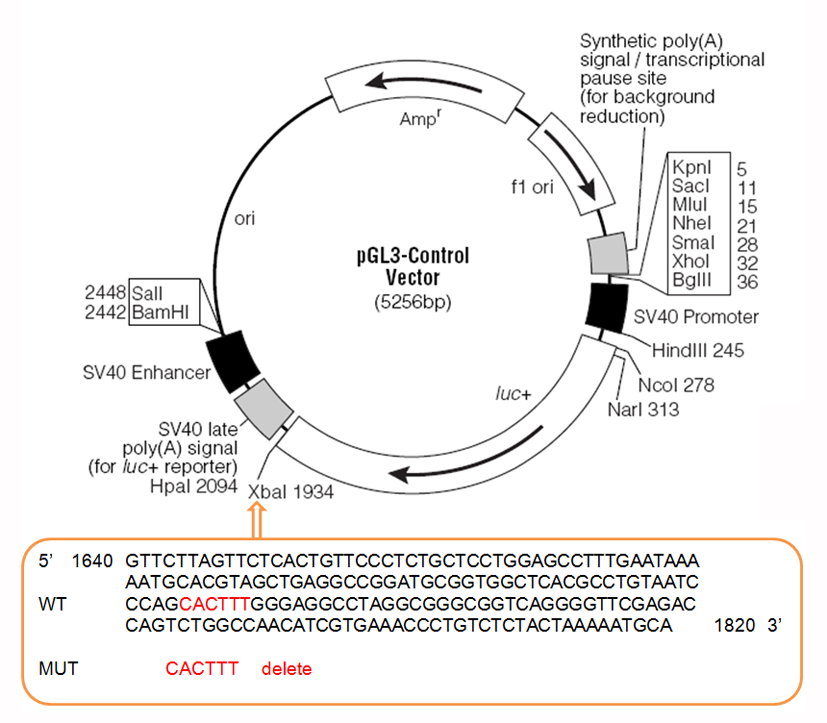

Supplement: Additional file 2: Figure S1 — The XbaI site of the pGL3-control vector (Promega). [file 1471-2407-13-478-S2.tiff]

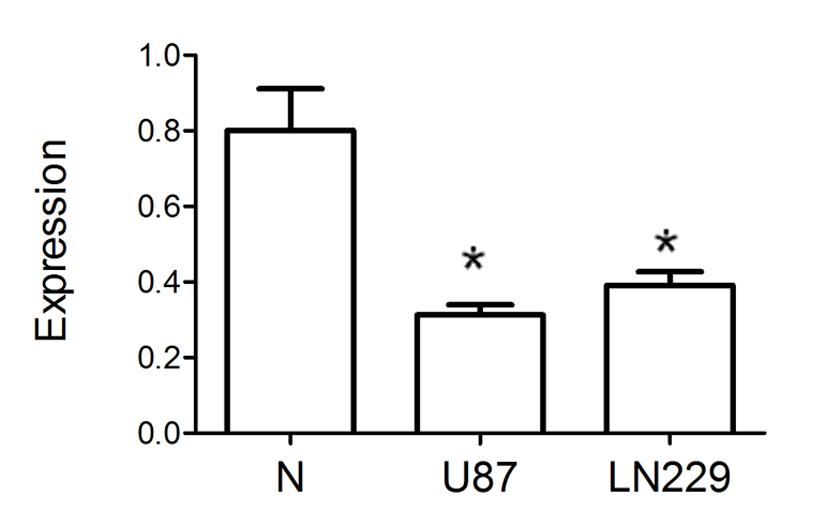

Supplement: Additional file 3: Figure S2 — miR-106a expression was significantly lower in U87 and LN229 cells. [file 1471-2407-13-478-S3.tiff]
